# A Foregut Duplication Cyst of the Stomach in Association with a Gastrointestinal Stromal Tumor and a Leiomyoma: A Case Report

**DOI:** 10.1155/2016/1537240

**Published:** 2016-12-21

**Authors:** Andréanne Gagné, Olga Sazonova, Simon Marceau, Martine Périgny, Philippe Joubert

**Affiliations:** ^1^Department of Pathology, Institut Universitaire de Cardiologie et de Pneumologie de Québec, Quebec City, QC, Canada; ^2^Department of Bariatric Surgery, Institut Universitaire de Cardiologie et de Pneumologie de Québec, Quebec City, QC, Canada; ^3^Department of Pathology, Hôtel-Dieu de Québec, Quebec City, QC, Canada

## Abstract

*Objectives*. Duplication cysts are rare benign lesions usually arising in the gastrointestinal tract. We report a case of a 52-year-old woman with an incidental gastric mass found on computed tomography during a pregraft workup for a familial cardiomyopathy.* Methods*. The mass was completely excised by partial gastrectomy and gross examination revealed a cystic lesion containing two small solid nodules in its wall. Microscopic evaluation and immunohistochemistry study were performed to further characterize the cyst and the nodules. A comprehensive literature review of the NCBI database PubMed was also carried out.* Results*. While the cyst was diagnosed as a foregut duplication cyst, the solid nodules proved to be concomitant gastrointestinal stromal tumor (GIST) and leiomyoma. Both morphologic features and immunohistochemistry stains, including CD117, smooth muscle actin, and CD34 supported the diagnosis. Clinical course was benign and the patient had no clinical evidence of relapse ten months following the surgical procedure. The literature search did not reveal any other published case of a foregut duplication cyst presenting in combination with a GIST and a leiomyoma.* Conclusions*. To our knowledge, this is the first case of a composite lesion comprising a foregut duplication cyst of the stomach along with a leiomyoma and a GIST.

## 1. Introduction

Gastrointestinal duplication cysts are rare tumors that can arise anywhere in the digestive tract [1]. Stomach cysts represent about 7% of all duplication cysts. They are more frequently found in paediatric patients than in adults. Those cysts are classified by their epithelium lining: bronchogenic, enteric, or oesophageal [2]. Bronchogenic cysts are lined by a pseudostratified columnar ciliated respiratory epithelium. Their pathogenesis is poorly understood but they are thought to form after abnormal detachment of the foregut between the fourth and the seventh week of embryogenesis [3]. Foregut duplication cysts are usually found in the supradiaphragmatic region (in the lung, at the hilum, or in the oesophagus). However, isolated cases of lesions developing in the subdiaphragmatic region have been reported, including in the stomach [4]. They seem to be mostly benign lesions, but exceedingly rare cases of thoracic bronchogenic cysts were found to be associated with malignant cells [5].

Mesenchymal tumors of the stomach are also infrequent neoplasms. Gastrointestinal stromal tumors (GIST) are the most common among them, with an incidence of 0,32 per 100 000 person-years [6]. Sixty percent of GIST are found in the stomach [7]. Although they usually present as incidental findings, some patients can present vague abdominal symptoms. Average age of presentation is between 60 and 70 years and there is no sex preference [8]. While GIST are more often low-grade tumors associated with an indolent clinical course, about 25% of them metastasize [7]. Histologic features such as tumor size and mitotic rate are important prognosis factors and the positivity for CD117 on immunohistochemistry is helpful to confirm the diagnosis.

Leiomyomas are benign neoplasms arising from the smooth muscle layer present along the gastrointestinal tract [3]. Gastric leiomyomas are rare neoplasms, about 50 times less frequent than GIST [7].

In the present report, we describe the case of a 52-year-old asymptomatic woman presenting a foregut duplication cyst of the stomach in association with a low-grade GIST and a leiomyoma.

## 2. Case Presentation

**Figure 1:**
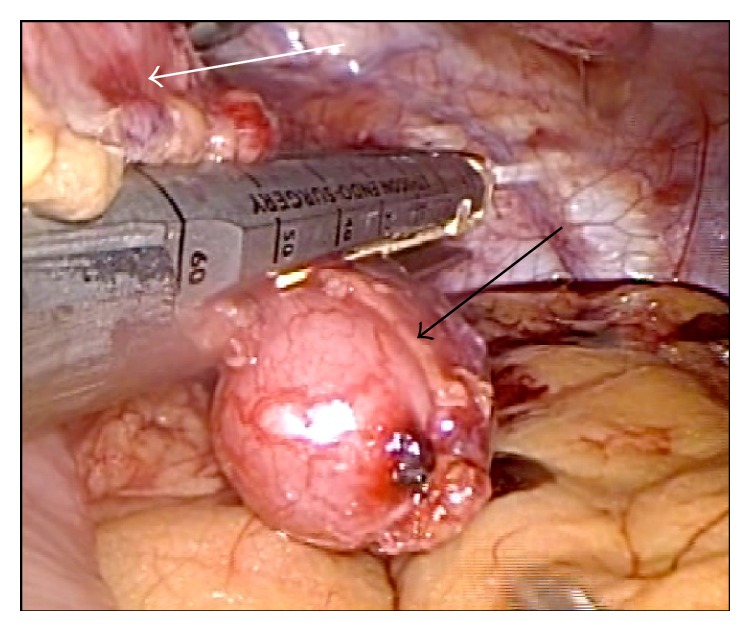
Gross image of the nodule (black arrow) during the surgical procedure. The white arrow points at the serosa of the stomach, from which the nodule was attached.

A 52-year-old woman known for a familial dilated cardiomyopathy was evaluated in the context of a pregraft workup. A chest CT scan was performed and a lesion was incidentally detected in the stomach next to the gastric fundus. The lesion was described as exophytic and suspicious but was otherwise difficult to characterize on imagery. The patient had no gastrointestinal clinical symptoms and the physical examination was normal. An endoscopic ultrasound (EUS) was done and showed a 2 cm hypoechogenic and well-encapsulated nodule that seemed to originate from the gastric muscularis. No adenopathy was identified. Because of the unknown nature of the lesion and the likely need of immunosuppressant medication in the future for cardiac graft management purpose, it was decided to remove the mass for further characterization. A laparoscopic partial gastrectomy was performed. A minimally adherent 2 cm lesion located at the gastric fundus near the gastroesophageal junction and the left crus of the diaphragm was completely removed and sent for pathology examination (see Figure 1).

### 2.1. Pathologic Findings

#### 2.1.1. Macroscopic Features

The resected specimen consisted of a small portion of the stomach measuring 3.7 × 2.5 × 2.0 cm and showing a 2.5 cm cystic lesion. The cyst contained a brownish mucoid substance and showed a smooth regular lining except for two distinct areas of nodularities measuring, respectively, 0.5 and 0.2 cm. The largest nodule was partly submitted for frozen examination and a diagnosis of low-grade spindle cell neoplasm was made.

#### 2.1.2. Microscopic Features and Immunohistochemistry

**Figure 2:**
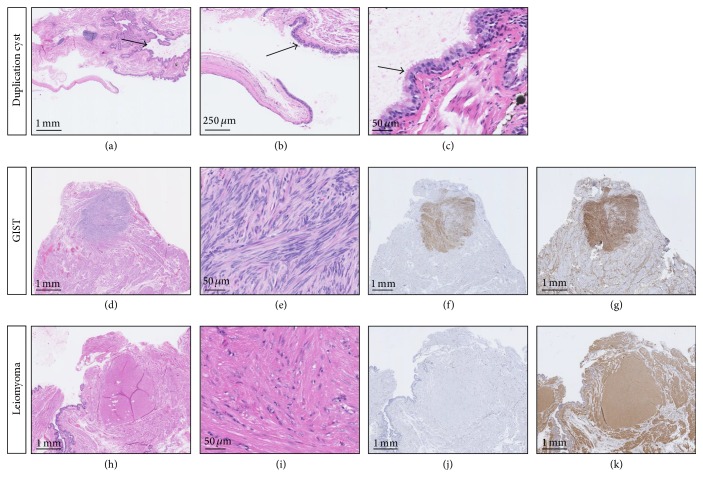
((a) and (b)) Duplication cyst at low power. The arrows point at the epithelial lining of the cyst, H&E. (c) Pseudostratified ciliated epithelial lining of the duplication cyst at high power. The arrow indicates the presence of cilia, H&E. ((d) and (e)) GIST nodule at low and high power. The tumor cells have spindle shape and mild cytologic atypia, H&E. ((f) and (g)) Immunohistochemistry stains show that the tumor cells are strongly positive for both CD117 (f) and CD34 (g). ((h) and (i)) Leiomyoma at low and high power. The tumor cells have elongated nuclei and abundant cytoplasm, with no cytologic atypia, H&E. ((j) and (k)) Immunohistochemistry stains show that the tumor cells are negative for CD117 (j), while they are strongly positive for SMA (k).

Histologically, the cystic lesion showed a benign pseudostratified ciliated epithelium consistent with a foregut duplication cyst of the stomach. The 0.2 cm nodule was histologically characterized by proliferation of plump spindle cells without significant mitotic activity (less than 5 mitoses/50 HPF). As illustrated in Figure 2, the tumor cells were labelled strongly and diffusely for CD117 (polyclonal rabbit anti-human CD117, Dako, California, USA) and CD34 (monoclonal mouse anti-human CD34 class II clone QBEnd-10, Dako, California, USA) but not for actin (monoclonal mouse anti-human muscle actin clone HHF35, Dako, California, USA), which was in agreement with a low-grade gastrointestinal stromal tumor (GIST). The second nodule had a 0.5 cm diameter and was characterized by bland-looking spindle cells with elongated and cigar-shaped nuclei. The lesion had strong and diffuse staining for smooth muscle actin (SMA), whereas CD34 and CD117 immunostainings were negative, consistent with a diagnosis of benign leiomyoma. While diffuse labelling for CD117 has been shown to be very specific for GIST, SMA positivity can be seen in both leiomyoma and GIST [9]. However, the strong staining seen for SMA combined with the absence of labelling for CD117 is consistent with a leiomyoma [10, 11]. Overall, these findings supported a diagnosis of a foregut cyst associated with a low-grade GIST and a leiomyoma. Following the initial histologic evaluation, consultation was requested to a gastrointestinal pathologist (MP) at the Hôtel-Dieu de Québec (Québec City, Québec, Canada). Eventually, the case was sent to Johns Hopkins Hospital (Baltimore, Maryland, USA) for further characterization. The initial diagnosis was confirmed.

### 2.2. Literature Review

In order to identify similar cases, an extensive literature review was performed using NCBI database PubMed in June 2015. The terms ((“Bronchogenic Cyst”[Mesh]) OR foregut cyst) AND (stomach) were used because the search using Mesh terms for bronchogenic cyst, leiomyoma, and GIST retrieved no results. The research was limited to English language. 70 citations were identified. Of those, 31 articles were available for reading and were retained. Five additional articles were identified in the references of the initially selected articles. A total of 36 articles with the description of 37 gastric foregut cyst cases were included in the present literature review. Thirty-seven cases of gastric foregut cyst were identified. None of them were associated with GIST or leiomyoma. Another literature review using the terms ((“Leiomyoma”[Mesh]) and (“Gastrointestinal stromal tumor”[Mesh])) showed one case of metachronous occurrence of GIST and leiomyoma at different locations. The summary of literature review is provided in Table 1 in the Supplementary Material available online at http://dx.doi.org/10.1155/2016/1537240.

## 3. Discussion

The present case report describes a very unusual location of foregut duplication cysts presenting in association with a GIST and a leiomyoma. To our knowledge, there is no known association between duplication cyst, GIST, leiomyoma, and idiopathic cardiomyopathy, as seen in our patient. Also, such a composite lesion has never been reported. The literature search for leiomyoma and GIST revealed only one published case of a 22-year-old girl with Carney triad who presented with metachronous gastric GIST and oesophageal leiomyoma [12]. Unlike our case, the two neoplasms were found in different locations and diagnosed two years apart. Furthermore, no foregut cyst was diagnosed in that case.

Thirty-seven cases of gastric foregut cyst have been identified in the literature review (Table 1, Supplementary Material). There were 21 women and 16 men (22 to 81 years old). While the vast majority of patients were asymptomatic, some were investigated for symptoms resulting from the compression of surrounding organs. The sizes of the lesions ranged between 1.6 and 40 cm and they were located anywhere in the proximal stomach. All of them presented a pseudostratified columnar ciliated respiratory epithelium and some showed other features of the normal bronchopulmonary tree such as cartilage and glands. In most cases, diagnosis was established after complete excision. Cytological examination of fine needle aspirates permitted accurate diagnosis in only three patients. There were no associated tumors found.

Foregut cysts are the result of abnormal pinching of the foregut during embryogenesis; however, their exact pathogenesis remains unknown [13]. They usually occur in the mediastinum and the lung parenchyma, but they also have been reported in the cutaneous and subcutaneous regions of the neck and oropharynx. Lesions are usually evaluated by computed tomography and endoscopic ultrasound [13].

## 4. Conclusion

In summary, we report an unusual case of a 52-year-old woman presenting a bronchogenic foregut cyst of the stomach in association with a leiomyoma and a GIST. To our knowledge, no similar cases have been reported. The present case provides new lines of evidence of neoplastic processes occurring within the bronchogenic cyst wall and contributes to the justification of their complete excision and of a thorough pathological examination.

## Supplementary Material

Comprehensive litterature review on gastric bronchogenic cysts using NCBI database PubMed.
