# Supplementary material for: A Foregut Duplication Cyst of the Stomach in Association with a Gastrointestinal Stromal Tumor and a Leiomyoma: A Case Report
Source: Case Rep Pathol. 2016 Dec 21;2016:1537240. doi: 10.1155/2016/1537240 (PMC5209591; doi:10.1155/2016/1537240)
Supplement: Supplementary file 1 — Comprehensive litterature review on gastric bronchogenic cysts using NCBI database PubMed. [file 1537240.f1.docx]

**Supplementary materials Table 1 – Literature review on foregut cysts**

| Author | Sex | Size | Clinical presentation | Localisation | Epithelium | Treatment | Follow-up |
| --- | --- | --- | --- | --- | --- | --- | --- |
| Keohane 1988 ([11](#_ENREF_11))  Braffman 1988 ([12](#_ENREF_12)) | 64 f | 15 | Abdominal pain, nausea and vomiting | Sub-diaphragmatic | Mature respiratory epithelium, submucous glands, smooth muscle, cartilage | Cystectomy | Post-op ok |
| Kim 2000([13](#_ENREF_13)) | 35 m | 7 | Epigastric discomfort | Lesser curvature | Pseudostratified columnar ciliated 60%, gastric 20% and erosion | Cystectomy | Post-op ok |
| Murakami 2008([14](#_ENREF_14)) | 72 f | 2 | Asymptomatic | Anterior wall of the middle third of the stomach | Pseudostatified ciliated et cuboid | Distal gastrectomy + lymph node dissection | 24 months uneventful follow-up |
| Yang 2013([15](#_ENREF_15)) | 50 m | 8 | Asymptomatic | Fundus and cardia | Pseudostratified ciliated | Cystectomy | 24 months uneventful follow-up |
|  | 37 f | 10 | Asymptomatic | Posterior wall | Pseudostatified ciliated and cuboid | Cystectomy | - |
| Cunningham 2006([16](#_ENREF_16)) | 63 f | 10 | Early satiety and abdominal pain | Fundus | Pseudostatified ciliated | Cystectomy | - |
| Khoury 2011([17](#_ENREF_17)) | 29 m | 8.5 | Abdominal pain | Fundus | Pseudostratified ciliated and gastric mucosa | Distal gastrectomy | - |
|  | 26 f | 5 | Abdominal pain, weight loss | Lesser curvature | Pseudostratified ciliated | Distal gastrectomy | - |
| Mardi 2010 ([18](#_ENREF_18)) | 42 m | 5.2 | Back pain | Cardia | Pseudostratified columnar ciliated | Cystectomy | Post-op ok |
| Theodosopoulos 2007 ([19](#_ENREF_19)) | 46 f | 8  3 | Loss of consciousness | Fundus and gastro splenic ligament | Pseudostratified columnar ciliated | Cystectomy + splenectomy | Post-op ok |
| Napolitano 2013 ([20](#_ENREF_20)) | 56 m | 5 | Asymptomatic | GOJ* | Pseudostratified columnar ciliated and cardial glands | Cystectomy | Post-op ok |
| Kurokawa 2013 ([21](#_ENREF_21)) | 71 m | 3 | Throat discomfort | Cardia | Pseudostratified ciliated | Distal gastrectomy | 12 months uneventful follow-up |
| Seddik 2012 ([22](#_ENREF_22)) | 40 m | 1.9 | Epigastric pain | Cardia | Diagnosis with scan | - | 72 months stability |
| Belli 2013 ([23](#_ENREF_23)) | 45 f | 8 | Abdominal pain, fatigue and weight loss | GOJ | Simple or pseudostratified columnar ciliated | Cystectomy | - |
| Jiang 2011 ([24](#_ENREF_24)) | 76 m | 4 | Asymptomatic | Lesser curvature | Pseudostratified columnar ciliated | Cystectomy | Post-op ok |
| Geng 2015 ([25](#_ENREF_25)) | 52 m | 6.5 | Epigastric discomfort | Lesser curvature | Simple columnar epithelium and pseudostratified columnar ciliated | Distal gastrectomy + lymph node dissection | 5 months uneventful follow-up |
| Sari 2012 ([26](#_ENREF_26)) | 22 m | 3 | Abdominal pain | Lesser curvature | Columnar ciliated | Cystectomy | Post op ok |
| Davenport 1999 ([27](#_ENREF_27)) | 13 f | 40 | Abdominal distension | Greater curvature | No epithelium apart from a few areas with plump, attened cells or foamy histiocytes | Cystectomy | - |
| Shireman 1987 ([28](#_ENREF_28)) | 61 f | 2 | Incidental at autopsy | Near GOJ | Pseudostratified columnar ciliated | - | - |
| Wakabayashi 2007 ([29](#_ENREF_29)) | 37 m | 5 | Abdominal pain and dysphagia | Lesser curvature | Pseudostratified ciliated | Cystectomy | Post op ok |
| Ballehaninna 2013([30](#_ENREF_30)) | 40 f | 5 | Dysphagia | Distal esophagus, sub-diaphragmatic | Ciliated | Cystectomy | 6 months uneventful follow-up |
| Rubio 2005 ([31](#_ENREF_31)) | 26 m | x | Epigastric pain | - | Pseudostratified cuboid | Fine needle aspiration | Post-op ok |
| Inaba 2010 ([32](#_ENREF_32)) | 64 f | 4 | Asymptomatic | Lesser curvature | Pseudostratified ciliated | Cystectomy | Post-op ok |
| Hedayati 2003 ([33](#_ENREF_33)) | 59 f | 7 | Asymptomatic | Posterior wall | Pseudostratified ciliated with gobelet cells. Mixed serous glands and cartilage | Cystectomy | - |
| Melo 2005 ([34](#_ENREF_34)) | 39 f | 6 | Asymptomatic | Fundus | Pseudostratified ciliated with focal immature squamous metaplasia | Cystectomy | Post op ok |
| Lee 2006 ([35](#_ENREF_35)) | 37 f | 7 | - | - | Pseudostratified ciliated | Cystectomy | - |
| Song 2005([36](#_ENREF_36)) | 62 f | 1.6 | Asymptomatic | Lesser curvature | Pseudostratified columnar ciliated, seromucinous gland and connective tissue, | Wedge resection | Post-op ok |
| Tan 2010 ([37](#_ENREF_37)) | 30 f | 5.1 | Asymptomatic | Posterior wall | Pseudostratified columnar ciliated | Cystectomy | Post-op ok |
| Laraja 1995 ([38](#_ENREF_38)) | 35 f | 5.5 | Epigastric pain and nausea | Greater curvature | Cuboidal to pseudostratified columnar ciliated | Cystectomy | - |
| Takahara 1996 ([39](#_ENREF_39)) | 25 m | 6.5 | Asymptomatic | Greater curvature | Pseudostratified columnar ciliated with focal squamous metaplasia and gastric epithelium with focal intestinal metaplasia | Cystectomy | - |
| Ikehata 2000 ([40](#_ENREF_40)) | 34 m | 10 | Asymptomatic | Greater curvature | Pseudostratified columnar ciliated and gastric foveolar epithelium | Cystectomy | 6 months uneventful follow-up |
| Sato 2008 ([41](#_ENREF_41)) | 60 f | 3 | Asymptomatic | Cardia | Cytology: ciliated columnar epithelial cells within a background of mucin | Fine needle aspiration | 24 months uneventful follow-up |
| Diaz Nieto 2010 ([42](#_ENREF_42)) | 67 m | 6 | Chronic low back pain | Lesser curvature | Ciliated and respiratory epithelium | Cystectomy | - |
| Ubukata 2011 ([43](#_ENREF_43)) | 81 f | 2.6 | Asymptomatic | Lesser curvature | Mature respiratory epithelium, glands, and smooth muscle | Cystectomy | 40 months uneventful follow-up |
| Hall 2007 ([44](#_ENREF_44)) | 40 m | 6.1 | Dyspnea, abdominal pain | Lesser curvature | Cytology: columnar ciliated cells | Fine needle aspiration | - |
| Kim 2015 ([45](#_ENREF_45)) | 43 f | 2.5 | Asymptomatic | Near GOJ | Respiratory | Wedge resection | - |
| Gensler 1966 ([46](#_ENREF_46)) | 46 f | 8 | Asymptomatic | Greater curvature | Pseudostratified columnar ciliated with focal squamous metaplasia | Cystectomy | Post-op ok |

* GOJ : Gastro-oesophageal junction
